# Supplementary figures and images for: The PP2A-like phosphatase Ppg1 mediates assembly of the Far complex to balance gluconeogenic outputs and enables adaptation to glucose depletion
Source: PLoS Genet. 2024 Mar 7;20(3):e1011202. doi: 10.1371/journal.pgen.1011202 (PMC10950219; doi:10.1371/journal.pgen.1011202)

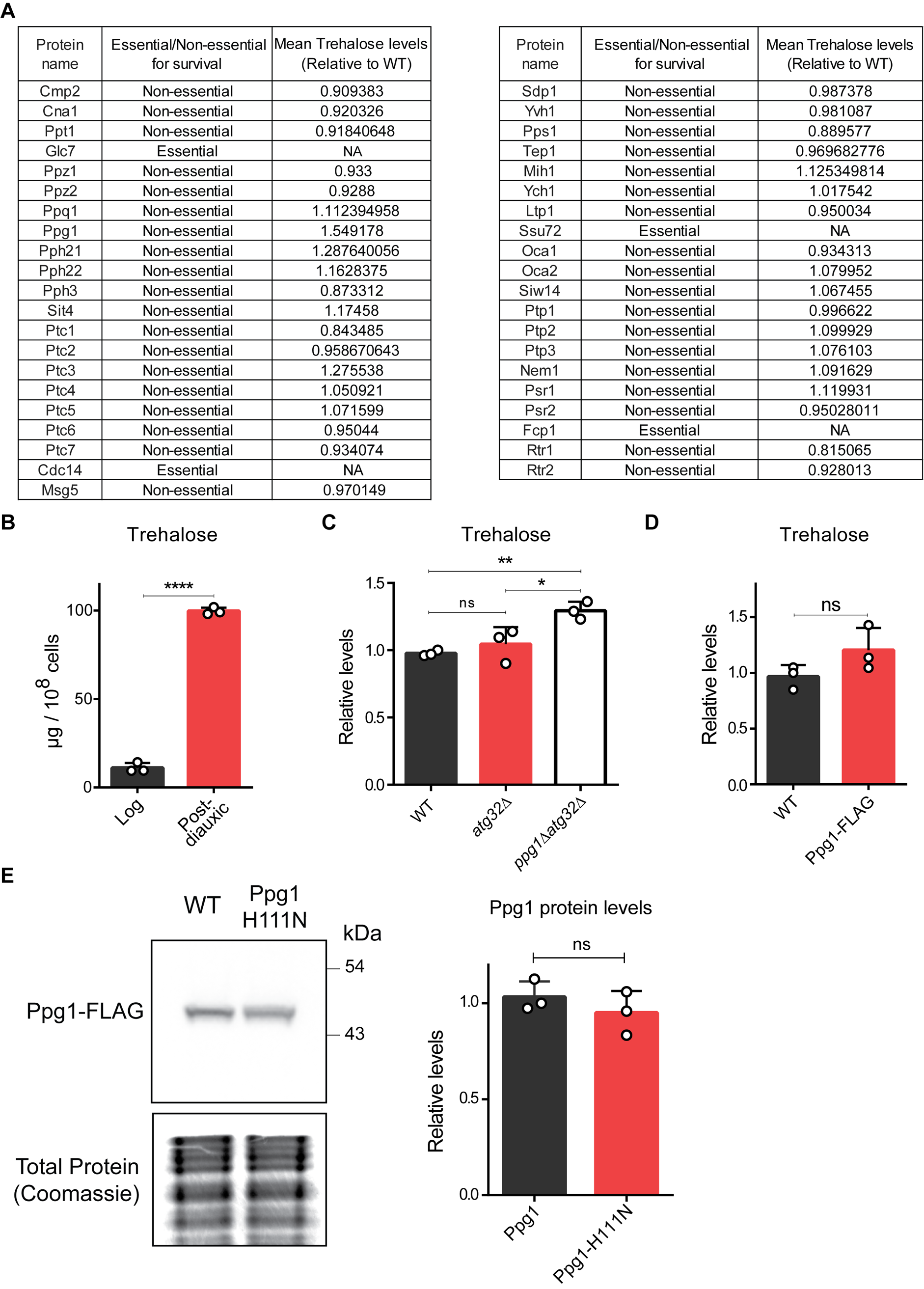

Supplement: S1 Fig — A) A list of protein phosphatases in S. cerevisiae and the phosphatase mutants used in the study. Trehalose accumulation was measured after 24hrs of growth in YPD medium. The mean trehalose accumulation was obtained from 2 biological replicates. B) Trehalose amounts in log and post-diauxic phase of growth. Trehalose levels were measured after 4hrs and 24hrs of growth in YPD medium. Data represented as a mean ± SD (n = 3). *P < 0.05, **P < 0.01, and ***P< 0.001; n.s., non-significant difference, calculated using unpaired Student’s t tests. C) Effect of deletion of Atg32 on trehalose accumulation in WT and ppg1Δ cells. Trehalose accumulation was measured after 24hrs of growth in YPD medium. Data represented as a mean ± SD (n = 3). *P < 0.05, **P < 0.01, and ***P< 0.001; n.s., non-significant difference, calculated using unpaired Student’s t tests. D) Relative trehalose levels in wild-type and Ppg1-FLAG cells after 24hrs of growth in YPD medium. Data represented as a mean ± SD (n = 3). *P < 0.05, **P < 0.01, and ***P< 0.001; n.s., non-significant difference, calculated using unpaired Student’s t tests. E) Effect of H111N point mutation on protein levels of Ppg1. The WT and Ppg1H111N cells containing endogenously tagged Ppg1 with 3xFLAG epitope were cultured in YPD medium. Cells were collected after 24hrs of growth and the levels of Ppg1 were measured by western blotting. A portion of the gel was Coomassie stained and used as a loading control. Western blot quantification was done using ImageJ software. A representative image is shown (n = 3). Quantification data represented as a mean ± SD (n = 3). *P < 0.05, **P < 0.01, and ***P< 0.001; n.s., non-significant difference, calculated using unpaired Student’s t tests. (TIF) [file pgen.1011202.s001.tif]

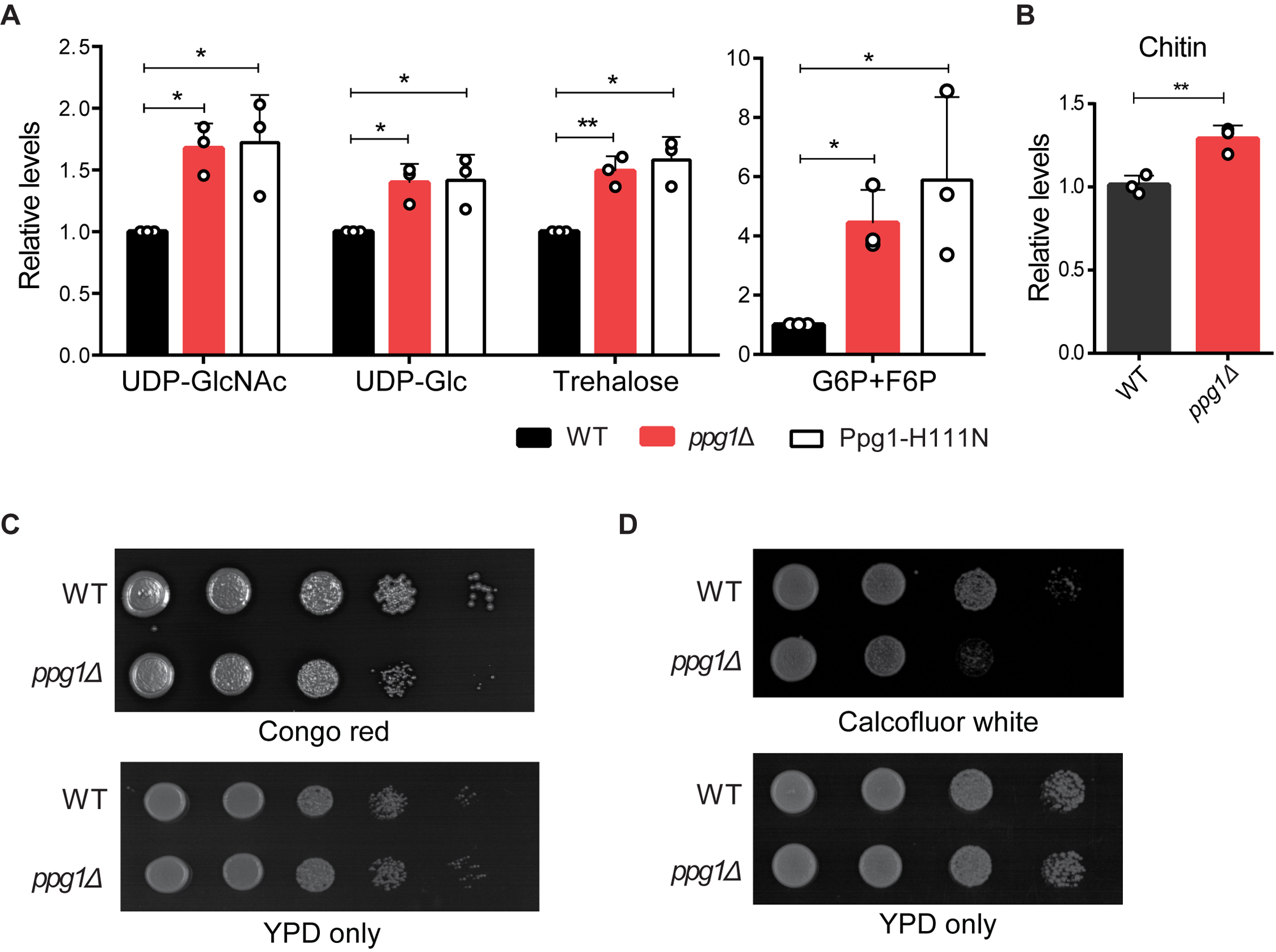

Supplement: S2 Fig — A) Relative steady-state amounts of specific gluconeogenic intermediates, precursors of cell wall and storage carbohydrates, and amino acids in WT, ppg1Δ, and Ppg1H111N cells after 24hrs of growth in YPD medium. Data represented as a mean ± SD (n = 3). *P < 0.05, **P < 0.01, and ***P< 0.001; n.s., non-significant difference, calculated using unpaired Student’s t tests. B) Relative chitin levels in cell walls of WT and ppg1Δ cells after 24hrs of growth in YPD medium. Data represented as a mean ± SD (n = 3). *P < 0.05, **P < 0.01, and ***P< 0.001; n.s., non-significant difference, calculated using unpaired Student’s t tests. C) The growth of WT and ppg1Δ cells in presence of Congo red. A serial dilution growth assay was carried out in presence of Congo red using WT and ppg1Δ cells. Congo red was used at a final concentration of 400 μg/ml. The images were taken after 60hrs of growth. A representative image is shown (n = 3). D) The growth of WT and ppg1Δ cells in the presence of Calcofluor white. A serial dilution growth assay was carried out in the presence of Calcofluor white. Calcofluor white was used at a final concentration of 50 μg/ml. The images were taken after 60hrs of growth. (TIF) [file pgen.1011202.s002.tif]

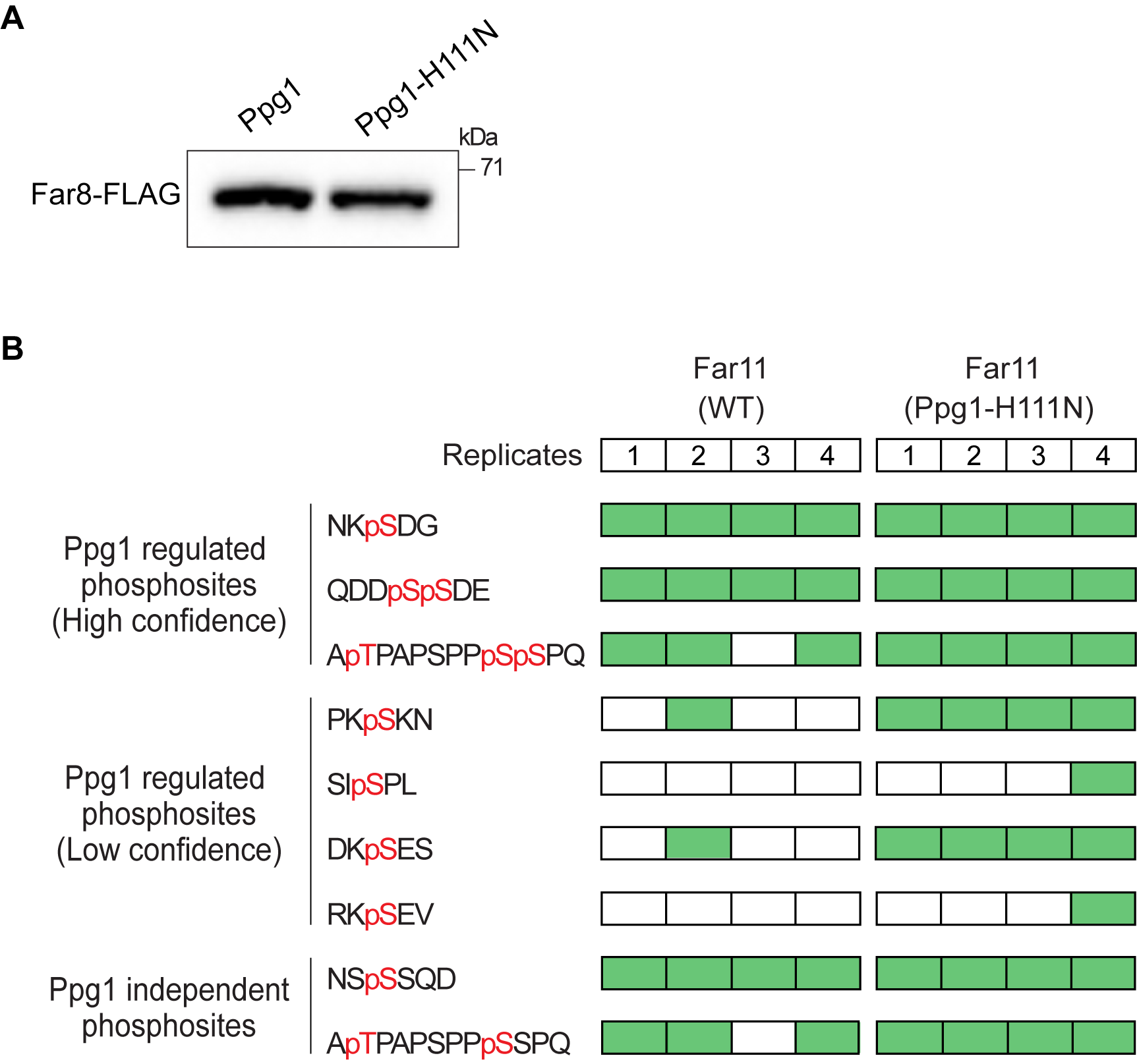

Supplement: S3 Fig — A) Regulation of Far8 post-translational modifications by Ppg1. WT and Ppg1H111N cells containing endogenously tagged Far8 with 3xFLAG epitope were cultured in YPD medium for 24hrs. Far8 mobility was monitored on a 7% SDS-PAGE gel. B) Schematic describing the Far11 phosphosites identified in various replicates of both wild-type and Ppg1H111N cells. The presence of a specific phosphopeptide in an individual replicate is denoted by a green box. (TIF) [file pgen.1011202.s003.tif]

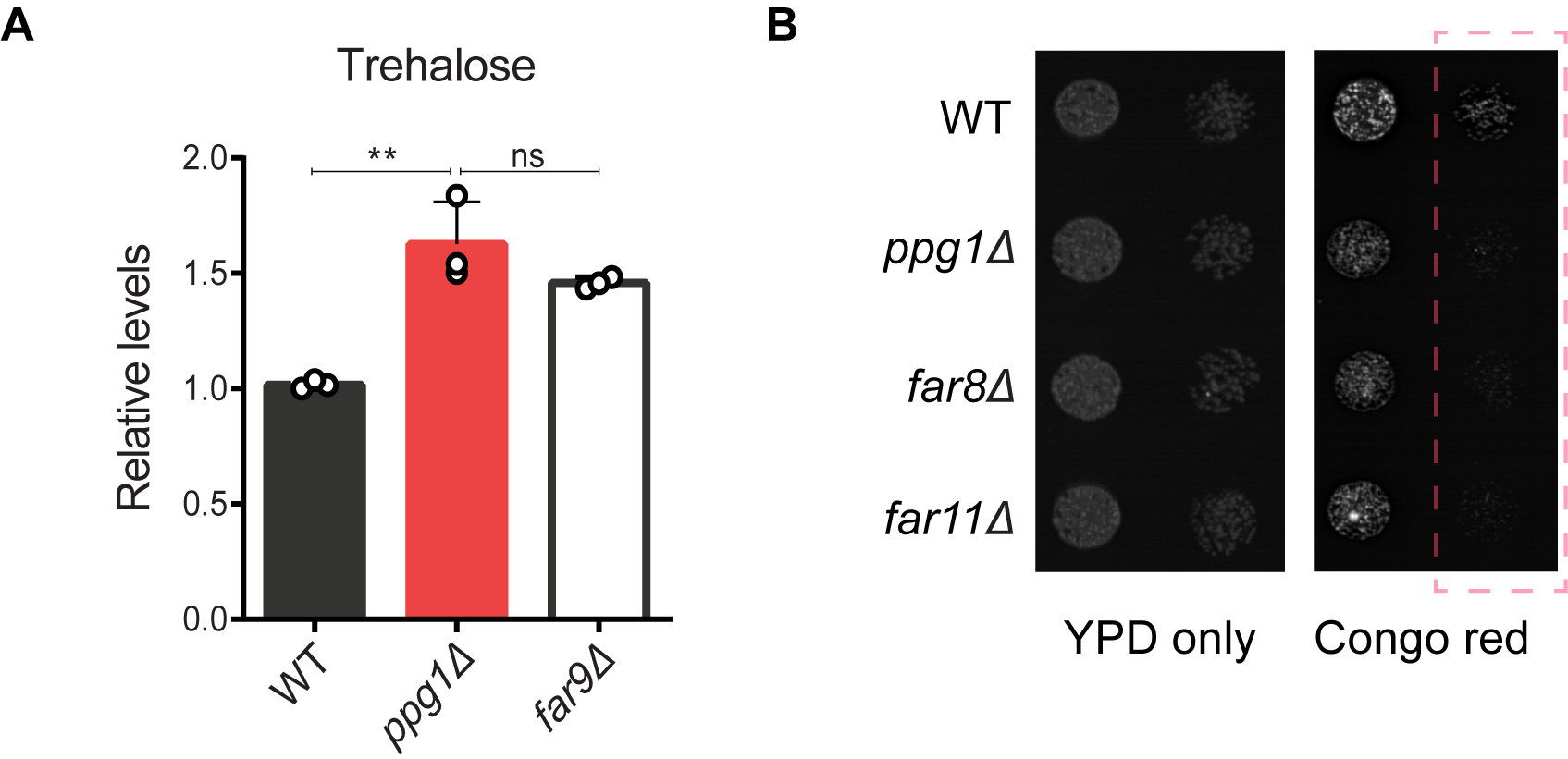

Supplement: S4 Fig — A) Relative trehalose levels in WT, ppg1Δ, and far9Δ cells after 24hrs of growth in YPD medium. Data represented as a mean ± SD (n = 3). *P < 0.05, **P < 0.01, and ***P< 0.001; n.s., non-significant difference, calculated using unpaired Student’s t tests. B) The growth of far8Δ, and far11Δ cells in presence of Congo red. A serial dilution growth assay was carried out in presence of Congo red using WT, ppg1Δ, far8Δ, and far11Δ cells. Congo red was used at a final concentration of 400 μg/ml. The images were taken after 60hrs of growth. A representative image is shown (n = 3). (TIF) [file pgen.1011202.s004.tif]

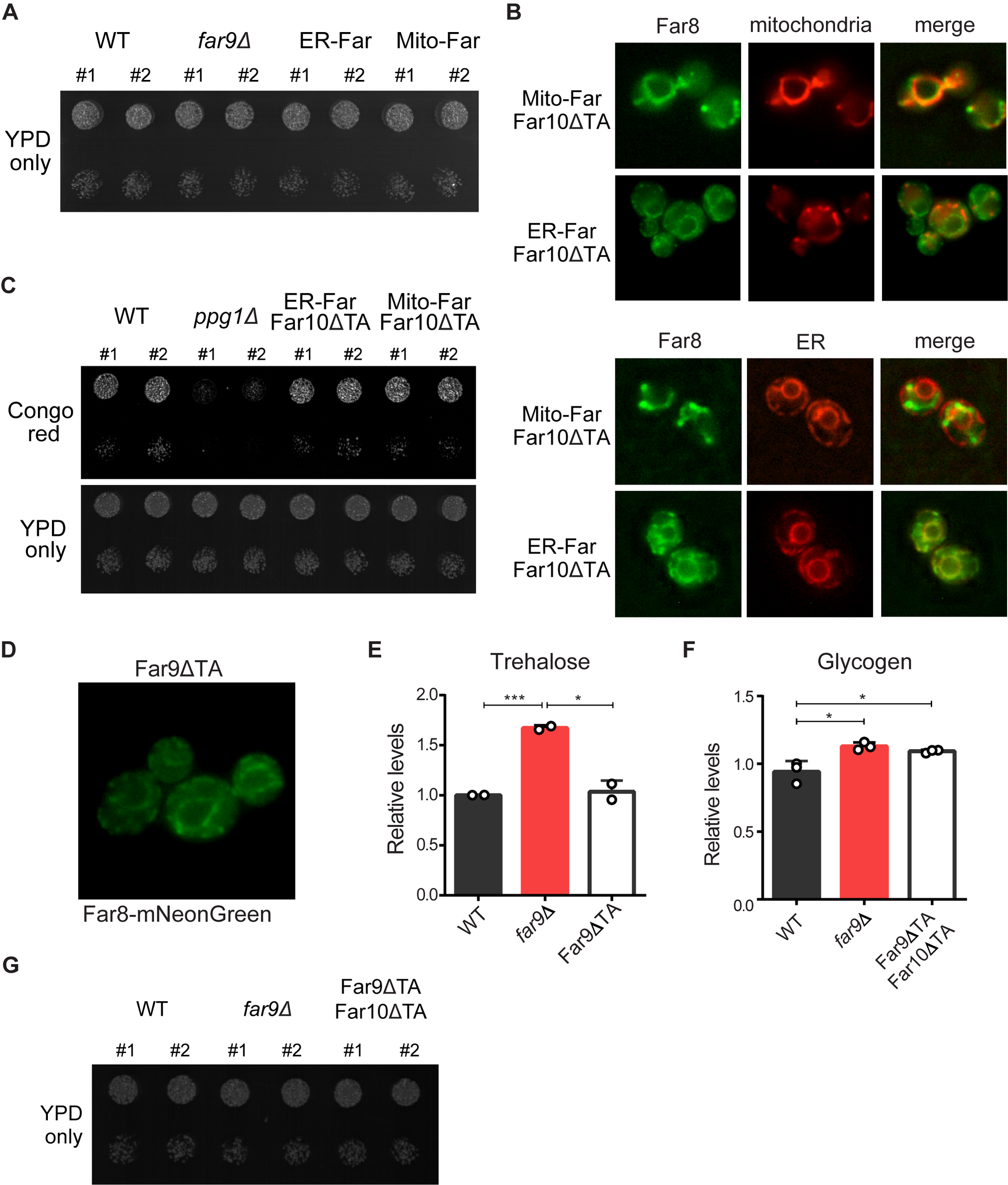

Supplement: S5 Fig — A) The growth of Mito-Far and ER-Far cells in YPD medium. A serial dilution growth assay was carried out using WT, Mito-Far, ER-Far, and far9Δ cells. The images were taken after 24hrs of growth. A representative image is shown (n = 2). B) The Mito-Far Far10ΔTA and ER-Far Far10ΔTA strains show distinct mitochondrial and ER localization of the Far complex. The Mito-Far and ER-Far cells were grown in YPD medium for 24hrs and analyzed by fluorescence microscopy. Far8-mNeonGreen was used to visualize Far complex. Sec63-mCherry was used to visualize ER. Mitochondria were visualized using MitoTracker red CMXRos. C) Growth of Mito-Far Far10ΔTA and ER-Far Far10ΔTA cells in presence of Congo red. A serial dilution growth assay was carried out in presence of Congo red using WT, Mito-Far Far10ΔTA, ER-Far Far10ΔTA, and ppg1Δ cells. Congo red was used at a final concentration of 400 μg/ml. The images were taken after 60hrs of growth. A representative image is shown (n = 2). D) The subcellular localization of Far complex in Far9ΔTA cells. Far8-mNeonGreen was used to visualize the localization of Far complex in these cells. E) Relative trehalose levels in WT, far9Δ, and Far9ΔTA cells after 24hrs of growth in YPD medium. Data represented as a mean ± SD (n = 2). *P < 0.05, **P < 0.01, and ***P< 0.001; n.s., non-significant difference, calculated using unpaired Student’s t tests. F) Relative glycogen levels in WT, far9Δ, and Far9ΔTA10ΔTA cells after 24hrs of growth in YPD medium. Data represented as a mean ± SD (n = 3). *P < 0.05, **P < 0.01, and ***P< 0.001; n.s., non-significant difference, calculated using unpaired Student’s t tests. G) The growth of Far9ΔTA10ΔTA cells in YPD medium. A serial dilution growth assay was carried out using WT, far9Δ, and Far9ΔTA10ΔTA cells. The images were taken after 24hrs of growth. A representative image is shown (n = 2). (TIF) [file pgen.1011202.s005.tif]

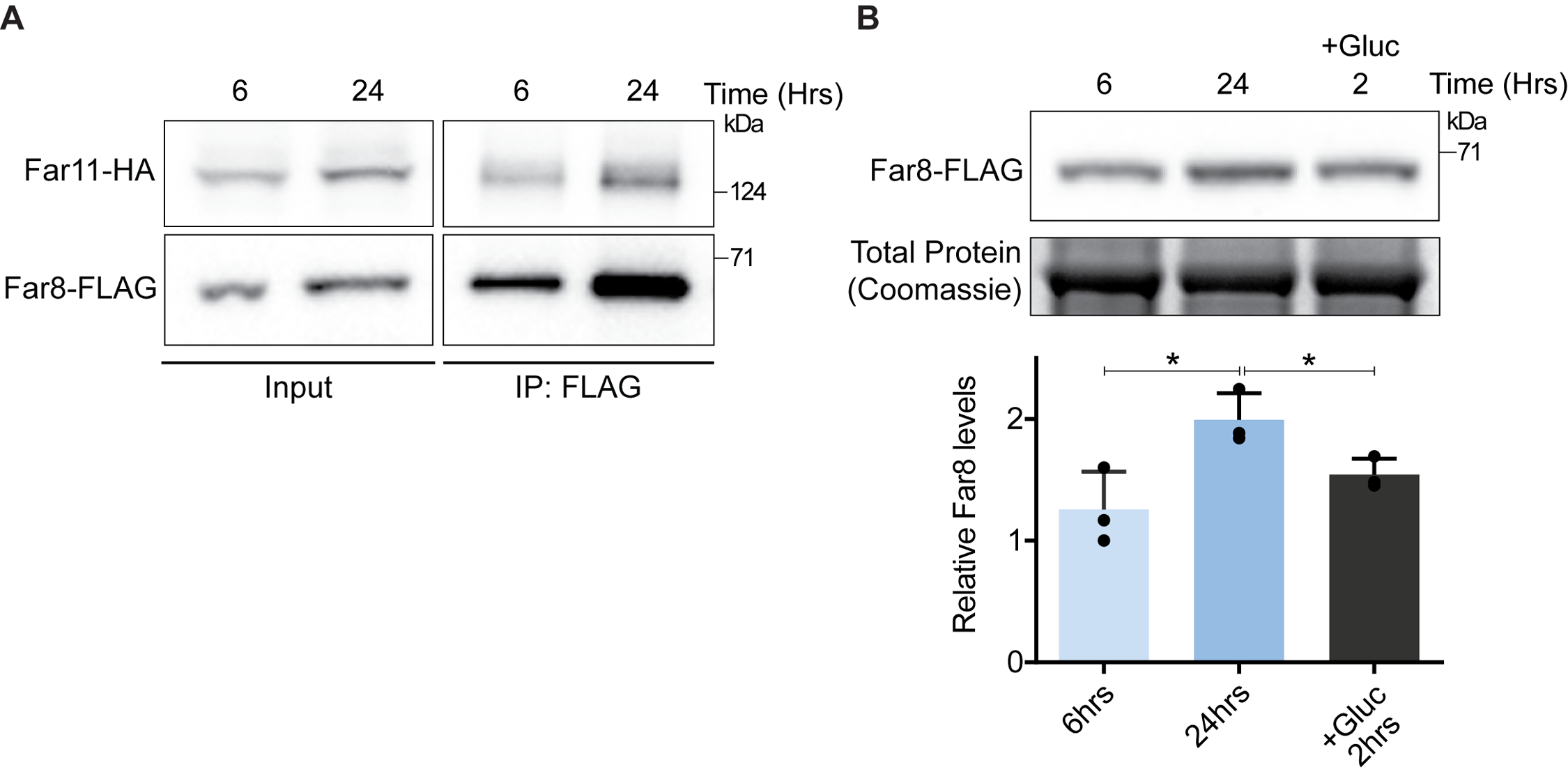

Supplement: S6 Fig — A) Interaction between Far8 and Far11 in different phases of growth. WT cells containing HA-tagged Far11 and FLAG-tagged Far8 were cultured in YPD medium. Cells were collected after 6hrs and 24hrs. Far8-FLAG was immunoprecipitated from these cells, and co-immunopurified Far11-HA was detected. A representative image is shown (n = 3). B) Effect of glucose availability on amounts of Far8. Cells expressing FLAG-tagged Far8 were cultured in YPD medium. After 24hrs of growth cells were diluted in the spent medium and 2% glucose was added. Cells were collected after 2hrs and levels of Far8 were measured by western blotting. A portion of the gel was Coomassie stained and used as a loading control. Western band quantification was done using ImageJ software. A representative image is shown (n = 3). *P < 0.05, **P < 0.01, and ***P< 0.001; n.s., non-significant difference, calculated using unpaired Student’s t tests. (TIF) [file pgen.1011202.s006.tif]

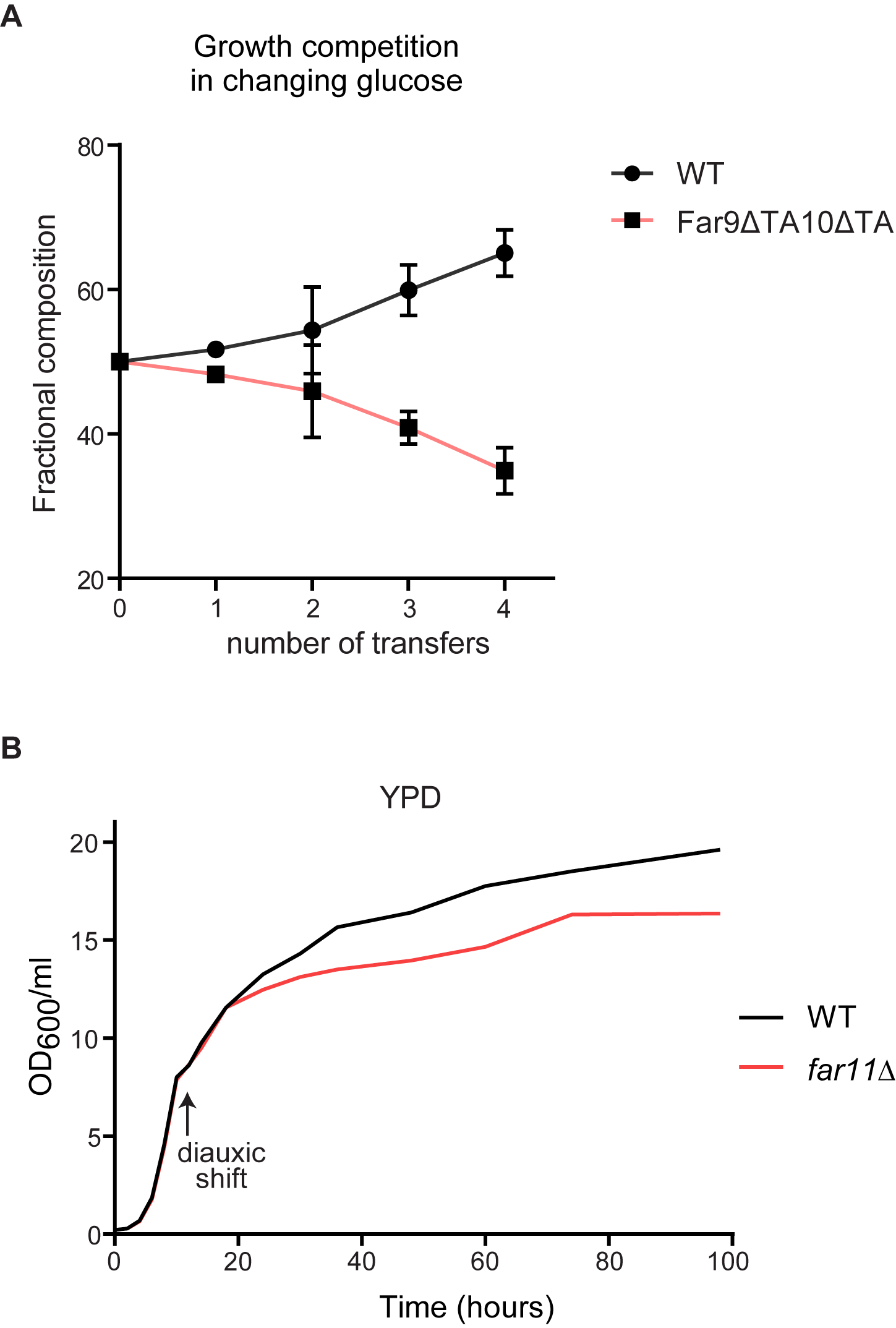

Supplement: S7 Fig — A) Competitive growth between WT and Far9ΔTA10ΔTA cells in changing glucose conditions. The total culturing time for the competition experiment is 96 hours. Data represented as a mean ± SD (n = 3). B) Comparative growth of WT and far11Δ cells in YPD medium. The cultures of WT and far11Δ were started at OD600 of 0.2 in YPD medium and the growth was monitored. (TIF) [file pgen.1011202.s007.tif]
