# Supplementary material for: The PP2A-like phosphatase Ppg1 mediates assembly of the Far complex to balance gluconeogenic outputs and enables adaptation to glucose depletion
Source: PLoS Genet. 2024 Mar 7;20(3):e1011202. doi: 10.1371/journal.pgen.1011202 (PMC10950219; doi:10.1371/journal.pgen.1011202)
Supplement: Attachment — Submitted filename: 137985_1_rebuttal_2837555_s3djh3.docx [file pgen.1011202.s013.docx]

To,

The editors,

Review Commons.

31^st^ Oct, 2023

We thank the editor and all the reviewers for the detailed critical feedback on our manuscript. We have substantially revised the manuscript to address all the queries, and have incorporated changes that address most of the suggestions made by the reviewers. The revised manuscript includes new experimental data, as well as text changes that address and clarify comments raised by the reviewers. The manuscript has been significantly strengthened by these revisions. A detailed response to reviewer comments is included below.

In the response letter below as well as the revised manuscript, we have addressed all the concerns raised by reviewers 1, 3, and 4, and most comments of reviewer 2. Some of the experiments suggested by reviewer #2 (related to an in-depth phosphoproteomics analysis) are unfortunately well beyond the scope of this manuscript, and infeasible at this stage (with the explanations provided below). We have divided our response document into three sections. In the first paragraph of the response, we briefly summarize the new data that we have included in the revision in response to reviewer queries. In the second section of this document, we have addressed the common comments raised by all the reviewers, which mostly address comments regarding the identification of Far11 phosphosites and mechanistic details about Far complex assembly. In the third part of this response, we include a detailed, point-by-point response to each of the reviewer's concerns, pointing to new data and specific changes made in the main manuscript. We also include a marked-up (in blue) version of the manuscript text, for easier follow up.

Our responses to queries are provided in blue text.

**Section I: A Brief summary of all new experimental data included in this manuscript**

- In this revised version of our study, we assessed the impact of the combined deletion of Ppg1 and components of Far complex. Our analysis revealed that double deletion of Ppg1 and Far complex does not result in any additive effect on metabolic regulation, indicating Ppg1 and Far function sequentially within the same pathway. Given these observations, the expectation will be that cells lacking Far complex will also have similar effects as the Ppg1 knock-out to adapt to glucose depletion. To assess this hypothesis, we conducted a competition experiment and indeed found that Far complex is required for proper adaptation to glucose depletion, very similar to Ppg1.
- To understand if Ppg1-Far regulate gene expression changes to modulate gluconeogenic outputs, we assessed transcript levels of gluconeogenic enzymes from *ppg1Δ* cells. Interestingly we observed that the transcript levels of these enzymes remain unchanged in *ppg1Δ* cells, suggesting mechanisms involving other modes of regulation.
- Additionally, our investigations revealed that the increased carbon allocation to cell wall precursors in *ppg1Δ* cells also makes them more susceptible towards the cell wall stress agent, Calcofluor white, similar to results obtained with Congo red.
- We have also carried out growth and competitive growth experiments with Far complex mutants, similar to those carried out with *ppg1Δ* cells, and find that these phenocopy *ppg1Δ* cells with respect to adapting to growth in glucose-depleted conditions.

**Section II: Regarding the identification of Far11 phosphosites dephosphorylated by Ppg1**

Our response to this concern:

We agree with the reviewers that in order to obtain the full mechanism of Far complex assembly, it will be important to identify the Far11 phosphosites regulated by Ppg1. However, current evidence already suggests that it will be very challenging to identify *specific* phospho-site targets of Ppg1. Currently available data from extensive phosphoproteomics studies in *Saccharomyces cerevisiae* have identified a very large number of phosphorylation sites on Far11 (Bodenmiller *et al*, 2010; Swaney *et al*, 2013; Soulard *et al*, 2010). The identified Ser/Thr residues on Far11 that are known to be phosphorylated are shown in the figure below, and include *at least 19 Ser/Thr residues*. Considering that so many Ser/Thr residues of Far11 are phosphorylated, it is hard to pinpoint specific phosphosites that are recognized and uniquely dephosphorylated by Ppg1. Additionally, it is widely established that Ser/Thr phosphatases, especially the PP2A family, have little selectivity. At least *in vitro*, as well as often *in vivo*, multiple PP2A family members non-selectively target the same residue. Therefore, most of the function is only revealed by a combination of genetic and biochemical data that identify contextual phenotypes (as we have done so, and established in this manuscript). At this stage of this study, these phosphoproteomics experiments are well beyond the scope of the current manuscript.

***Identified Ser/Thr residues known to be phosphorylated in only Far11***

**However, we acknowledge this important point raised, and now include it in the discussion in Line 507.**

**The revised text now reads:**

*“Additionally, large-scale studies suggest over 19 putatively phosphorylated Ser/Thr residues in Far11 alone, indicating multiple kinase-phosphatase interactions on this protein* (Bodenmiller et al, 2010; Swaney et al, 2013; Soulard et al, 2010)*. Phosphoproteomics experiments with Ppg1 mutants are therefore a good starting point, but in themselves may be insufficient to specifically identify Ppg1 specific phosphosites on Far11.”*

**We also now have an extensive discussion section included, on the challenges of identifying specific phosphatase sites on proteins (and the contrast with kinase dependent phosphorylation sites). This section reads as (Line 500):**

*“Separately,  phosphoproteomics-based studies could provide avenues for identifying as yet unidentified substrates of Ppg1. However, phosphoproteomics approaches have been far more suited for elucidating kinase-mediated regulation, due to high substrate specificity of kinases (Li et al, 2019). Identifying the specific substrates of phosphatases has posed significant challenges because of the nature of phosphatases like PP2A, which exhibit low substrate specificity and often have overlapping and compensatory outputs (Virshup & Shenolikar, 2009; Millward et al, 1999). Hence, determining the specific outputs or substrates of phosphatases through these methods presents a formidable challenge. Additionally, large-scale studies suggest over 19 putatively phosphorylated Ser/Thr residues in Far11 alone, indicating multiple kinase-phosphatase interactions on this protein* (Bodenmiller et al, 2010; Swaney et al, 2013; Soulard et al, 2010)*. Phosphoproteomics experiments with Ppg1 mutants are therefore a good starting point, but in themselves may be insufficient to specifically identify Ppg1 specific phosphosites on Far11.”*

**Section III: Point-by-point response to all individual reviewer comments:**

**Reviewer #1:**

The authors study metabolic adaptation to glucose depletion in budding yeast. A non-essential protein phosphatase mutant screen reveals adaptation to glucose depletion (growth in post-diauxic phase) requires Ppg1. The authors show i) that, in post-diauxic phase, cells lacking Ppg1 accumulate more trehalose, glycogen, UDP-glucose, UDP-GlcNAc (i.e., gluconeogenic outputs) than wild-type cells, ii) that, in post-diauxic phase, cells expressing a catalytically inactive version of Ppg1 accumulate more trehalose and iii) that Ppg1 is required for adaptation and growth post-glucose depletion. The authors find that Ppg1 interacts with Far11 (a member of the Far complex) in cells growing in the post-diauxic phase and that Ppg1 promotes Far complex stability. Finally, the authors conclude that the Ppg1 promotes Far complex stability to maintain gluconeogenic outputs after glucose depletion.

We thank the reviewer for a careful reading of this manuscript, and many constructive comments.

Major comments:

1) Figures 1 to 4. The authors show that loss of Far components phenocopies loss of Ppg1 and conclude that Ppg1 is upstream of Far. However, the authors do not determine the combined effect of the two mutations. The authors should assess the phenotype (e.g., gluconeogenic output levels) of cells lacking both Ppg1 and Far (or in far9_deltaTA far10_deltaTA cells lacking Ppg1). The authors' conclusion would be strengthened if there was no additive effect between the mutations.

Thank you for raising this point. We have now investigated the combined effect of deletion of Far11 and Ppg1 on post-diauxic carbon metabolism by measuring trehalose amounts. From these measurements, we did not observe any *additional* increase in trehalose amounts in the cells lacking both Ppg1 and Far11. We also assessed the growth of these cells in the presence of the cell wall stress agent, Congo red. There was no additional growth defect in the double deletion strain in the presence of Congo red. This data strongly indicates that the double deletion of Ppg1 and Far11 does not have an additive effect, indicating that both proteins function in the same pathway. **This data is now included in Fig. S3 D and E. Text changes are made accordingly in the results section line 300:**

*“To determine if Ppg1 and Far complex independently regulate carbon metabolism or function within the same pathway, we generated double deletion mutants of Ppg1 and Far11 (ppg1Δfar11Δ). We assessed trehalose accumulation in ppg1Δfar11Δ cells and found no additional increase in trehalose levels (Figure 3H). Furthermore, we studied the growth of these ppg1Δfar11Δ* cells *in the presence of Congo red and observed no additional growth defects (Figure 3I). Overall, these findings strongly indicate that Ppg1 and Far complex function sequentially within the same pathway, with Ppg1 upstream of Far.”*

Data showing trehalose levels from WT, *far11Δ* and *ppg1Δfar11Δ* cells after 24hrs of growth in YPD medium (now new Figure 3H):

Data showing the growth of WT, *far11Δ* and *ppg1Δfar11Δ* cells in the presence of Congo red (now new Figure 3I):

2) Related to Figure 6A-C. One would expect that cells lacking Far components (or far9_delta TA far10_deltaTA cells) show a similar phenotype (fail to adapt to growth in changing glucose compared with wild-type cells) as cells lacking Ppg1. Is this the case?

We agree with this expectation. The cells with loss of components of Far complex fully phenocopy *ppg1Δ* cells, and have an imbalanced carbon metabolism. Therefore, the expectation is that these cells will exhibit similar defects as *ppg1Δ* to properly adapt to glucose depletion. To address this question, we carried out a competition experiment with wild-type and Far9ΔTAFar10ΔTA cells (where the Far complex can now no longer be anchored, and therefore assemble properly, as shown in Fig. 4F, G), specifically assessing adaptation to environments as glucose depletes (and done identically to those with *ppg1Δ*). Note: the choice of this strain was to enable quantitative estimation, since we needed strains that had a ‘fluorescence’ mark (mNeonGreen or mCherry) to quantitatively assess changes in each genotype. Similar to *ppg1Δ* cells, the relative proportion of  Far9ΔTAFar10ΔTA cells decreased during the competition experiment (Fig S6A). Independently, we estimated changes in post-diauxic growth of *far11Δ* cells, starting in glucose-rich conditions. In batch culture, the *far11Δ* cells showed reduced growth specifically in the post-diauxic phase (Fig S6B). Effectively, the loss of the Far complex nearly perfectly phenocopies the loss of Ppg1 in enabling effective adaptation to glucose-depleting environments. This data reiterates the importance of the Far complex in adaptation to glucose depletion, as the mechanistic target of Ppg1 function. **This data is now included in the new Fig. S6 A and B. The text changes are made accordingly (Line 423):**

***“****To concurrently address the role of Far complex in enabling cells to adapt to glucose depletion, we carried out a similar competition experiment (as with ppg1Δ) with WT and Far9ΔTA10ΔTA cells. Note: the Far9ΔTA10ΔTA cells will not allow the Far complex to anchor and assemble within cells, as shown earlier, and therefore phenocopies far9Δ, and was utilized in this experiment for easier quantitative estimations based on fluorescence. Expectedly, the relative proportion of Far9ΔTA10ΔTA cells decreased during the course of the competition experiment (Figure S6A). We next examined the effect of loss of Ppg1 on steady-state batch culture growth, starting from a glucose-replete medium. The loss of Ppg1 did not affect growth in the glucose-replete log phase, but after cells entered the post-diauxic (glucose-depleted) phase, ppg1Δ cells showed reduced growth and a reduction in biomass accumulation (Figure 6D). Independently, we assessed the growth of far11Δ cells starting in glucose-replete conditions, and observed reduced growth of these cells specifically in the post-diauxic phase (Figure S6B), similar to ppg1Δ cells. Effectively, the loss of Ppg1 or the Far complex phenocopied each other, and collectively, these data reveal that Ppg1-Far mediated regulation enables adaptation and competitive growth fitness after glucose depletion.****”***

Data showing growth competition between WT and Far9ΔTA10ΔTA cells in changing glucose conditions (now new Fig. S6A):

Data showing growth dynamics of WT and *far11Δ* cells in the YPD medium (now new Fig. S6B):

3) The manuscript would be considerably strengthened if the authors provided more information on the mechanism by which Ppg1 controls Far complex stability, e.g., can the authors about the phosphosite(s) in Far11 regulated by Ppg1? As the authors mention, it has been already suggested that Ppg1 is required for Far complex assembly (PMID: 33317697).

This comment has been commonly addressed in the section 2 of this document. Briefly, while we are equally excited about this direction, given the complexity of phosphorylation of the Far11 protein (and the challenges specifically in the context of PP2A family phosphatase action), this component is likely to take years to address and is beyond the scope of this manuscript. We do include a more speculative section in the discussion in this regard.

Minor comments:

1) The authors may consider to include data from Figures 6A, 6B and 6C (failure to adapt to glucose-changing conditions) after Figure 2 to show a complete characterization of the phenotype of cells lacking Ppg1. Figure 6 could show only the "proposed model".

We appreciate the reviewer’s suggestion and had indeed considered this possibility in an early version of the writing of the manuscript. However, we prefer the current flow of this manuscript, where we identify Ppg1 as a putative regulator of gluconeogenic flux, and end with the mechanistic confirmation of function that links Ppg1 and Far complex to the same adaptation function. We hope the reviewer appreciates this viewpoint.

2) Related to Figure 1. The authors mention that Ppg1 is a "notable hit" and that the "increase in post-diauxic trehalose levels are considerable". However, there is no reference to use as a comparison. Is there any other mutant strain known to accumulate trehalose at the post-diauxic shift? If yes, it would be informative if the authors compared the effect of such mutant strain to a ppg1-delta mutant.

For this experiment, we did not employ another mutant as a reference for increased trehalose accumulation. However, the point raised by the reviewer in itself was interesting in itself. Looking into the literature, we find that there are no reliable, quantitative estimates of how much trehalose increases in yeast in the post-diauxic phase (compared to the log phase), although numerous manuscripts (including some of our own earlier work) allude to this point. Therefore, we did this experiment, to obtain absolute quantitative information on the trehalose amounts in YPD in cells after 4 hrs of growth in 2% glucose, vs. in cells the post-diauxic phase (24 hrs after starting growth in 2% glucose). The amounts of trehalose increase >10-fold in the post-diauxic phase compared to the log phase. **We now mention this in the text, and include absolute quantitation of trehalose amounts in Fig S1B.** Hence, a ~1.5-fold further increase in trehalose amounts *in the post-diauxic* *ppg1Δ* cells compared to post-diauxic wild-type cells is considerable. **The revised text now reads (Line 121):**

*“We initially estimated how much trehalose amounts increased in the post diauxic phase. Trehalose amounts increased over 10-fold in the post-diauxic phase after 24 hours of growth starting in 2% glucose, compared to cells after 4 hours of growth in the same condition (Figure S1B).”*

*“Compared to the ~10 fold increase in trehalose (as shown in Figure S1B), the further increase of 1.5-fold in trehalose accumulation in the post-diauxic phase in cells lacking Ppg1 (Figure 1D) is substantial.”*

Data showing trehalose accumulation in log and post-diauxic phase wild-type cells (Figure S1B):

3) Figures 4D and 4F. Regarding sensitivity to Congo Red and compared to wild-type cells, it seems that cells lacking Far9 are much more sensitive to Congo Red in Figure 4F than in Figure 4D. Is this just an image quality issue? The authors should address this apparent discrepancy.

The small visual difference is likely because the images (which come from experiments done at different times) were taken at slightly different time points after spotting. To avoid any confusion, in the figure legends we have now mentioned the precise time at which each of the images were taken.

Reviewer #2:

Summary:
In this manuscript, the authors screened the yeast phosphatase mutant that shows defective in metabolic adaptation and found that PP2A-like phosphatase Ppg1 is required for the appropriate gluconeogenic outputs after glucose depletion. Furthermore, they showed that Far complex which assembles with Ppg1 is also required to maintain gluconeogenic outputs. They also found that Ppg1 is required for assembly of Far complex and the assembly on the ER or mitochondrial membrane is important for their function. Ppg1 and Far complex dependent control of gluconeogenic outputs had important role on adaptive growth under glucose depletion.

Major comments:

In this study, the authors report new evidence that the Ppg1 and Far complexes are involved in the regulation of gluconeogenic outputs. However, the mechanism by which the Ppg1-Far complex is involved in gluconeogenic outputs has not been fully analyzed, and further analysis of the role of Ppg1 in Far complex assembly and the significance of Far11 phosphorylation is needed. The authors should consider the following points,

We thank the reviewer for valuable, constructive comments. Investigating the mechanism through which Ppg1, via the Far complex, regulates gluconeogenesis outputs and unravelling the added mechanism of Far complex assembly in this context are exciting areas of future research, and indeed where we hope to go. However, at this stage addressing some of these follow-up questions is beyond the scope of this manuscript. Our current findings unambiguously identify Ppg1 as a phosphatase that controls post-glucose depletion *gluconeogenic flux*, also identifies this mechanism to function through the proper assembly of the Far complex, and show that cells function through a Ppg1 - Far axis to adapt to glucose depletion. At this stage, we do not know what the Far complex might help assemble, and while this is an obvious follow-up, we anticipate years of effort to unravel this next question.

1) In the mutant screen, both pph21Δ and pph22Δ cells showed increased levels of trehalose (figure 1C). Pph21 and Pph22 are catalytic subunits of protein phosphatase 2A (PP2A) and function redundantly. Thus, it may be possible that PP2A is more involved in gluconeogenic outputs regulation than Ppg1.

In *S. cerevisiae*, the PP2A phosphatases regulate phosphorylation of transcription factors that control storage carbohydrate synthesis, and thereby regulate carbon metabolism (Bontron *et al*, 2013; Clotet *et al*, 1995; Dokládal *et al*, 2021). Notably, the deletion of Pph21 and Pph22 results in *increased transcription* of glucose repressed genes (Castermans *et al*, 2012), consequently resulting in increased gluconeogenesis and storage carbohydrate synthesis in these mutants. Additionally, our screen data also found an increase in trehalose accumulation in mutants of Pph21 and Pph22 (which were less than that of the Ppg1 mutants). Collectively, these observations emphasize the role of PP2A phosphatases in regulating gluconeogenic outputs, primarily through transcriptional control.

In notable contrast to the transcriptional changes observed with Pph21/22 mutants, the Ppg1-mediated regulation described in this manuscript does not involve any transcriptional changes of the enzymes of gluconeogenesis and related carbon metabolism (**now included in Fig 2E** ). This excitingly points towards regulation by some combination of post-translational modifications, allostery, or mass action. In light of these disparities, we believe that the function of Ppg1 elucidated in this study, operates independently of PP2A-mediated regulation of carbon metabolism. This point is now **included in the discussion (Line 456).** **The revised text now reads:**

*“There are well studied examples of signaling systems regulating metabolic adaptation, which have typically focused on understanding the repression or activation of relevant transcriptional outputs. For example, upon glucose depletion, the Snf1 kinase activates transcription factors such as Cat8 and Rds2, resulting in an increase in transcripts of key gluconeogenic enzymes (Turcotte et al, 2010; Rashida et al, 2021; Vengayil et al, 2019). In this context, phosphatases belonging to PP2A family, particularly Pph21 and Pph22, regulate transcriptional outputs of glucose repressed genes (Bontron et al, 2013; Castermans et al, 2012). Interestingly, and in contrast to this, the Ppg1-mediated regulation we uncover in this study does not rely on changes in gene expression (Fig. 2E). Instead, this points towards regulation through other mechanisms that are driven by post-translational modifications, mass action, or enzyme concentration etc. This function of Ppg1, as uncovered in this study, differs from regulation mediated by related phosphatases.”*

2) Is it the Far complex or Ppg1 activity that is required for the regulation of gluconeogenic outputs? It seems that assembly of the Far complex requires Ppg1 and Ppg1 activity requires the Far complex. However, either one should be involved in the regulation of gluconeogenic outputs. For example, Innokentev et al, 2020 concluded that Ppg1 activity is critical for the regulation of mitophagy and that the Far complex serves only as a scaffold for Ppg1. by Ppg1 dephosphorylating an unidentified protein. The possibility that Ppg1 may be involved in the regulation of glycolytic output by dephosphorylating unidentified substrates needs to be fully tested.

We agree with the reviewer's point. Our data very clearly now demonstrate that the Ppg1 activity is required for the assembly of Far complex (Fig. 3D). Subsequently, our data shows that the Far complex is required for regulation of gluconeogenic outputs. These observations together suggest the following two hypotheses:

1. Ppg1 is required for assembly of the Far complex. The assembled Far complex could transiently interact with other proteins that regulate gluconeogenic outputs (as would be possible for a ‘scaffolding system’). Ppg1 is primarily required for the assembly of the Far complex, and may not directly regulate signaling proteins that control gluconeogenesis.

2. Alternately, the Far complex only serves as a scaffold, and enables Ppg1 to interact and dephosphorylate as yet unidentified substrate(s).

These two possibilities are also not mutually exclusive. Both these possibilities merit further investigation, but at this stage, all our direct biochemical experiments (including isolating the Far complex and Ppg1, in order to identify interacting proteins) have not yielded more than this connection between Ppg1 and Far itself. Given this (and what would be a reasonable expectation for a dynamic scaffold), it is likely that the subsequent targets of Ppg1 and Far are transient interactions. A future effort would involve creating proximity-based target identification systems in *S. cerevisiae* (that work effectively in glucose-depleted conditions) and identifying such mechanisms.  Currently, no such system exists, and we are building these kinds of tools for future studies. Exploring such mechanisms of gluconeogenic outputs is therefore a very interesting area of future investigation, but well beyond the scope of this manuscript. **We include an acknowledgement of the same in the discussion section (Line 490). The revised text now reads:**

***“****Notably, the Ppg1 phosphatase regulates post-diauxic carbon metabolism by modulating the assembly of the Far complex (Fig. 3). Considering this requirement of Ppg1 to assemble this scaffolding complex and thereby constrain gluconeogenic flux, our study presents two intriguing possibilities: first, the Far complex scaffold could act as a facilitator, enabling interaction between Ppg1 and its other substrates (which regulate gluconeogenic outputs); and second, the primary function of Ppg1 is to facilitate Far complex assembly, which transiently brings to proximity other signaling proteins and enzymes that control gluconeogenesis. Both these possibilities (which are not mutually exclusive) merit detailed investigation. However, exploring these would require the development of new, proximity-based target identification systems for yeast that can identify transient protein-protein interactions. Separately,  phosphoproteomics-based studies could provide avenues for identifying as yet unidentified substrates of Ppg1. However, phosphoproteomics approaches have been far more suited for elucidating kinase-mediated regulation, due to high substrate specificity of kinases (Li et al, 2019). Identifying the specific substrates of phosphatases has posed significant challenges because of the nature of phosphatases like PP2A, which exhibit low substrate specificity and often have overlapping and compensatory outputs (Virshup & Shenolikar, 2009; Millward et al, 1999). Hence, determining the specific outputs or substrates of phosphatases through these methods presents a formidable challenge.****”***

3) Although there are no known substrates of Ppg1 other than Atg32, Atg32 is not involved in the regulation of gluconeogenic outputs. The identification of substrates of Ppg1 involved in the regulation of gluconeogenic outputs will help to elucidate the molecular mechanism of gluconeogenesis.

We completely agree with the reviewer's point (and also see our response to the previous point). It’s important to note that the involvement of Ppg1 in regulating mitophagy is entirely independent of its role in regulating gluconeogenic outputs. This is something we firmly establish in this study, in Fig. S1C. This indicates that in addition to its recognized role in Atg32 dephosphorylation specific to extreme starvation conditions of mitophagy, Ppg1 activity functions to regulate gluconeogenesis, a critical homeostatic function. Our response to the previous comment indicates our future lines of inquiry, which are currently well beyond the scope of this manuscript. **Included in the discussion (Line 500). The revised text now reads:**

***“****Separately,  phosphoproteomics-based studies could provide avenues for identifying as yet unidentified substrates of Ppg1. However, phosphoproteomics approaches have been far more suited for elucidating kinase-mediated regulation, due to high substrate specificity of kinases (Li et al, 2019). Identifying the specific substrates of phosphatases has posed significant challenges because of the nature of phosphatases like PP2A, which exhibit low substrate specificity and often have overlapping and compensatory outputs (Virshup & Shenolikar, 2009; Millward et al, 1999). Hence, determining the specific outputs or substrates of phosphatases through these methods presents a formidable challenge.****”***

4) The authors conclude that Ppg1 dephosphorylates Far11 and that dephosphorylated Far11 assembles with the Far complex. However, there is a possibility that Ppg1 activity is required for Far complex assembly independently of dephosphorylation of Far11. To prove the authors' assertion, it is necessary to identify the phosphorylation site of Far11 and show that its phosphorylation affects the binding of Far11 to Far8.

We address this point in the earlier section 2 of this document and hope that the reviewer will recognize the extreme challenges in the feasibility of these experiments at the current stage

5) Several kinases have been reported to be involved in gluconeogenic outputs regulation. The initial aim of this study was to identify phosphatases involved in gluconeogenic outputs regulation by antagonizing these kinases. However, Ppg1 has not been shown to be involved in transcriptional regulation to control carbon metabolism by antagonizing any kinase.

We thank the reviewer for raising this important point, which is one of the highlights of the findings of this manuscript. The regulation of gluconeogenesis enzymes by dephosphorylation/phosphorylation is not yet known at all, nor has there been a prior reason to look for such regulation. This paper will now provide strong reasons to look for such regulation. Separately, almost all past effort has been to look at transcriptional responses to changes in carbon availability. This is despite overwhelming evidence (Hackett *et al*, 2016) that over 50% of metabolic regulation in yeast can be direct - at the level of flux regulation by mass action, substrate availability and/or allostery- and precludes transcriptional changes. Since this interesting point was raised, we compared the expression of transcripts of the enzymes of gluconeogenesis, storage carbohydrate metabolism and cell wall synthesis in wild-type and *ppg1Δ* cells. Notably, we did not observe significant changes in transcript levels of these enzymes in *ppg1Δ* cells. This provides additional evidence that suggests the regulation of gluconeogenic *flux* (which we quantitatively demonstrate in Fig 2) must be via alternate mechanisms that involve increasing local substrate concentrations/PTMs/enzyme scaffolds or other mechanisms that need not invoke transcription. We therefore believe that it will be very interesting to study these possibilities in the future, and this can lead to a rich line of future inquiry. Our study also opens the possibility that Ppg1 might counteract kinase-mediated signaling (which, in the context of glucose, is better studied with PKA, TORC1 and other outputs). **We have now included the transcript analysis of gluconeogenic enzymes in WT and *ppg1Δ* cells, now included in the new Fig 2E.**

**We also include this revised text, to reiterate this point (Line 212):**

*“Finally, we asked if Ppg1 regulates the expression of transcripts of enzymes involved in gluconeogenesis, storage carbohydrate metabolism and cell wall synthesis proteins to modulate gluconeogenic outputs. For this, we measured the transcript levels of these enzymes from post-diauxic WT and ppg1Δ cells. Notably, the transcript levels of these enzymes remain unchanged in ppg1Δ cells (Figure 2E). This data suggests that Ppg1-mediated carbon flux regulation does not involve any transcript level changes, indicating that Ppg1 regulates gluconeogenic flux via mechanisms that involve allosteric, post-translational or mass action-based regulation.”*

Data showing transcript levels of enzymes of gluconeogenesis, storage carbohydrate metabolism, and cell wall synthesis proteins in WT and *ppg1Δ* cells (now new Fig. 2E):

We also include a few lines in discussion, where we reiterate that while much of our understanding of the regulation of gluconeogenesis comes from changes in transcriptional programs, substantial regulation of metabolic flux involves direct regulation via allostery, post-translational modifications, mass action and concentration (Hackett *et al*, 2016). Ppg1, via the Far complex, appears to participate in one such example of regulation (Line 463).

*“the Ppg1-mediated regulation we uncover in this study does not rely on changes in gene expression (Fig. 2E). Instead, this points towards regulation through other mechanisms that are driven by post-translational modifications, mass action, or enzyme concentration etc. This function of Ppg1, as uncovered in this study, differs from regulation mediated by related phosphatases. How might this occur? An underappreciated but important mediator of metabolic adaptation is the direct modulation of metabolic outputs or flux, through a combination of mass action and allosteric regulation (and without invoking transcriptional changes). Even in unicellular organisms like S. cerevisiae, over 50% of metabolic regulation occurs through such mechanisms (Hackett et al, 2016).”*

6) If the assembly of the Far complex is involved in gluconeogenic outputs regulation, what is the mechanism? The Far complex is a scaffold for enzymes. Therefore, the role of the Far complex in gluconeogenic outputs regulation will not be elucidated until the enzymes that function there are identified.

We agree that the specific mechanism of how the Far complex functions, after being assembled by Ppg1, cannot be understood unless we find those targets. Indeed, the Far complex may potentially interact with signaling proteins or metabolic enzymes involved in post-diauxic carbon metabolism. However, many of these interactions are transient and identifying these interactions is an extremely challenging task. As pointed earlier, we will now have to establish effective methods in yeast for proximity-based substrate/target identification, establish effective mass spectrometry-based pipelines for the same, and then screen for new regulators. This is by no means a trivial task, and is well beyond the scope of this manuscript, and we hope the reviewer recognizes the same.

**Recognizing this point, we had mentioned this in the discussion (Line 553):**

**“***In order to understand how dynamically assembled scaffolds with varying localizations and modifications can regulate homeostatic outputs such as metabolic adaptations, we require new chemical biology approaches that stabilize low-affinity protein-protein interactions, or substrate-trapping mutants to identify transient substrates that are brought together by such signaling hubs (Qin et al, 2021). This remains a key challenge in the context of protein phosphatases, which naturally interact with substrates with low affinities (Bonham et al, 2023).***”**

Minor comments:

1) Because TA of Far10 can tether Far complex on the membrane, Mito-Far and ER-Far experiments (Figure 4A-D) should be performed under Far10ΔTA conditions.

We agree with the reviewers' comment. In the Mito-Far and ER-Far cells, the Far10 protein (with intact TA domain) can localize to the surface of both organelles. Our careful microscopy images show clear localization/targeting of components of Far complex to respective compartments in both Mito-Far and ER-Far cells. This data strongly indicates that regulating localization of Far9 by itself (at either surface location) is sufficient for Far complex to localise to these compartments.

2) Figure 6B and 6C, total culturing time (hours) should be shown on X-axis in addition to number of transfers.

We now include this in the figure legends.

3) Figure 3E, additional explanation is needed as to why the molecular mobility of Far11-FLAG after CIP treatment differs between Ppg1-H111N and Ppg1.

The difference in mobility of Far11 in wild-type and Ppg1-H111N cells can be because of post-translational modifications other than phosphorylation. However, these modifications are regulated in a Ppg1-dependent manner. It will be interesting to identify these modifications on Far11 and their role in stabilizing the Far complex assembly. **We now include this in the text (Line 285):**

*“At this stage, while these experiments are consistent with a role of dephosphorylation in Far11 function and the assembly of the Far complex, these do not preclude other post-translational modifications in addition to phosphorylation.”*

Reviewer #3:

The study performed by Niphadkar et al. seeks to uncover the role of the phosphatase Ppg1 in regulating gluconeogenesis during post-diauxic shift in S. cerevisiae. The authors show that loss or inactivation of Ppg1p affects production of gluconeogenic products incl. trehalose and glycogen. The authors show that assembly of the Far complex required the activity of Ppg1 and is required to maintain gluconeogenic

outputs after glucose depletion.

The manuscript is clearly written and methods well considered, no omics-methods have been included. Especially phosphoproteomics would be relevant to include. Specifically, the tracing experiments are an interesting and appropriate approach to confirm effects on gluconeogenesis etc. Yet, working with regulation of posttranslational modifications (phosphorylations) it is surprising that the authors only to a limited extent examine phosphorylation events, and not all examine or discuss specific phosphorylation events of e.g. Far11.

The study is interesting and provides new insights into regulation of glucose metabolism in yeast, however, there are serious concerns that need to be addressed before it can be reconsidered for publication.

Major points:

1) The authors use electrophoretic mobility assays w/wo CIP to address the phosphorylation state of Far11. They show in figure 3E that the mobility of Far11 depends on Ppg1 activity and can be affected by CIP. Why is the mobility of Far11 not affected in e.g. figure 3D?

For these experiments, protein gels with different acrylamide concentrations were used. For the shift experiment, proteins were resolved using 7% gel for the duration of 5 hours. In Fig. 3D, 4-12% gradient gels were used and proteins were resolved for the duration of 2 hours. **We now mention this in the figure legends and methods section.**

2) There are several sites in Far11 previously reported to be phosphorylated, see e.g. Bodenmiller et al 2010 (Science Signal.) Are there sites that are specifically regulated (dephosphorylated) by Ppg1? or by other phosphatases? kinases?

This is addressed in section 2 of this document. In that section, we summarize the very large number of putative Ser/Thr residues that are phosphorylated in Far11, and while there is no clear information on which kinases might act on these, it is extremely complex to identify specific phosphatase roles in dephosphorylating these.

3) Here, it would be appropriate to apply phosphoproteomics to examine Far11 phosphorylation in Ppg1 knock out cells or in cells with inactivated Ppg1.

We agree with the reviewer's comment. It will be very interesting to implement phosphoproteomics to identify phosphosites regulated by Ppg1. However, unlike kinases, the changes in the phosphoproteome with phosphatase knock-outs are very challenging to interpret, especially for a family of phosphatases from the PP2A family. Due to a range of overlapping substrate recognition sites, as well as a change in kinase outputs when a phosphatase is missing, interpreting phosphoproteomes with phosphatase knockouts, which function conditionally (during say post-diauxic conditions, like this study), will have substantial challenges. See for example this very recent, exhaustive, state-of-the-art study in yeast, quantifying kinase and phosphatase mutant phosphoproteomes (Li *et al*, 2019). While the analysis for kinase mutants were substantially more revealing, the data from phosphatase mutants were very convoluted, and could identify very little specific outputs of phosphatase function. This set of experiments is beyond the scope of this manuscript, but this manuscript provides compelling reasons to do so. We have included a couple of lines in the discussion, related to this specific component. **Included in the discussion (Line 500).**

*“Separately,  phosphoproteomics-based studies could provide avenues for identifying as yet unidentified substrates of Ppg1. However, phosphoproteomics approaches have been far more suited for elucidating kinase-mediated regulation, due to high substrate specificity of kinases (Li et al, 2019). Identifying the specific substrates of phosphatases has posed significant challenges because of the nature of phosphatases like PP2A, which exhibit low substrate specificity and often have overlapping and compensatory outputs (Virshup & Shenolikar, 2009; Millward et al, 1999). Hence, determining the specific outputs or substrates of phosphatases through these methods presents a formidable challenge. Additionally, large-scale studies suggest over 19 putatively phosphorylated Ser/Thr residues in Far11 alone, indicating multiple kinase-phosphatase interactions on this protein (Bodenmiller et al, 2010; Swaney et al, 2013; Soulard et al, 2010). Phosphoproteomics experiments with Ppg1 mutants are therefore a good starting point, but in themselves may be insufficient to specifically identify Ppg1 specific phosphosites on Far11.”*

4) The authors show that the levels of Ppg1 remain constant during growth in YPD medium, while the levels of Far11 increased after 24hrs of growth in YPD medium, and thus argue that the amount of Far complex itself increases in post-diauxic phase. The authors need to show that the level of complex indeed increases.

In addition to Far11, we also compared the amounts of Far8 - another core component of Far complex. Similar to Far11, we observed an increase in the amounts of Far8 specifically in the post-diauxic phase. We also assessed the amounts of Far8 in response to glucose availability, and find that Far8 also decreases when glucose is added to the system. These data support our findings that the amounts of Far complex increase in the post-diauxic phase. **These data are included in Fig. S5 B.**

In the text, we reiterate (Line 396):

*“Furthermore, the amounts of Far8 also were reduced after addition of glucose to post-diauxic cells (Figure S5B). Together, we infer that the activity and amounts of Ppg1 are constitutive, but the amounts of the Far proteins are glucose-responsive (Figure 5E).”*

Data showing the effect of glucose availability on Far8 protein amounts are included in (Fig. S5B):

5) The authors also apply fluorescence microscopy to address the localization of the Far11 complex etc. The quality of the shown images should be improved, also merged images should be shown. Only one single image containing one cell is shown, images should ideally show additional cells in the same image, alternatively, additional images should be shown.

**Good point. We have now included higher quality images which show more cells in each frame, as well as include the merge/overlap, in Fig. 4B.** This should satisfy concerns.

For the reviewer’s reference,  some additional images  is are shown here:

Reviewer #4:

General comments

This paper reports a critical function of PP2A-like phosphatase encoded by PP1G in the post-diauxic shift of the yeast Saccharomyces cerevisiae. This function is mediated via the assembly of FAR complex that naturally sites at the ER-mitochondria outer membranes to ensure proper onset of growth at the diauxic shift by appropriate carbon allocation through gluconeogenesis. The identification of this PPase was based on a screen of yeast mutant defective in non-essential PPases for trehalose accumulation.

This is a bit surprising as it is known that trehalose accumulation sets in as soon as glucose is depleted and continues steadily during growth on other carbon source, which is merely ethanol, although it may depend whether the experiment was carried out in YPD or in mineral synthetic medium as YNB.

Although the work seems experimentally well conducted, in particular for the demonstration that bPP1G interacts with FAR complex, it raises several issues requiring a thorough revision and additional experiments to truly support the role of PP1F in regulating post-diauxic shift.

1) all experiments were done using YPD medium and only a single value of trehalose at 24 h was recorded! It will be important to ensure that all mutant had exactly same growth rate, that at 24 h, glucose was totally gone. It should be relevant to have a more complete kinetic analysis of trehalose/ glycogen accumulation along growth, monitoring as well glucose consumption in WT and the pp1gD, to really convince that the metabolic difference is not associated with a difference in glucose consumption, such as that at 24 h, there is still some glucose remaining in the culture of the mutant!

We thank the reviewer for raising these interesting points. We address this comment in following two points:

1. In Fig.2 C, we have shown the kinetics of trehalose accumulation during the course of growth. Additionally, we measure amounts of other gluconeogenic outputs as cells grow and deplete glucose. Only after cells deplete glucose and enter the post-diauxic phase do we observe an increase in amounts of these metabolites in *ppg1Δ* cells.

2. We measured extracellular glucose concentration at 24hrs, and there was no detectable glucose present in the medium. This indicates that cells have completely consumed available glucose and have shifted to gluconeogenic metabolism. **We now mention this in the text (Line 117):**

*“For the screen output, trehalose accumulation was assessed from these mutants after 24hrs. At this 24hrs time point, no glucose was detected in the medium, confirming that cells are in the post-diauxic phase. Trehalose synthesis increases in the post-diauxic phase and is a reliable readout of a gluconeogenic state.”*

Note for further references: in several earlier studies, we have systematically established that trehalose production is a very reliable indicator of *gluconeogenic flux* (e.g. see PMID: 32876564, PMID: 31758251, PMID: 27090086, and PMID: 31241462). In addition, we have extensively described ways to look at trehalose production in this methods paper PMID: 32181267.

Finally, we will note that there are very few studies that actually have estimated *flux* through gluconeogenesis, and have made most inferences using only the expression of transcripts of gluconeogenic genes, and hence the interpretations have to be made accordingly. Our study, to our knowledge, is the first to provide quantitative carbon flux measurements through gluconeogenic intermediates, in the context of *any phosphatase mutant* studied in yeast. All other studies have not measured flux, but rely on changes in transcripts, or steady-state amounts of storage carbohydrates to draw conclusions.

2) The experiment at least with pp1gD and WT should be redo in mineral synthetic medium with 2% glucose. There, only ethanol can be the sole carbon for growth resumption and thus this will ensure that the effects is linked to growth resumption at diauxic growth as YPD is a rich medium that contains excess of many amino acids and peptides that may interfere with your phenotype.

We have examined the growth of *ppg1Δ* cells in a synthetic medium with glucose as the sole carbon source. These data indicate that *ppg1Δ* cells grow similar to wild-type cells in the log phase (glucose-replete). However, these cells show reduced growth post-glucose depletion, where the only available carbon source is secreted ethanol. Here, as expected in a synthetic minimal medium, the extent of difference in growth of *ppg1Δ* cells is not as pronounced as seen in YPD medium as the spent post-diauxic medium here may not provide enough carbon source to fuel further growth.

Data showing the growth dynamics of *ppg1Δ* cells in SD medium:

Additionally, to study the role of Ppg1 in regulating post-diauxic metabolism in cells growing in SD medium, we measured amounts of gluconeogenic outputs from the post-diauxic cells. Notably, after 18 hours of growth in the SD medium, the *ppg1Δ* cells showed increased amounts of gluconeogenic outputs (UDP-GlcNAc, F16BP). Collectively, this data suggests that Ppg1 regulates gluconeogenic outputs in cells growing in a synthetic medium with glucose as the only carbon source.

Data showing the relative levels of UDP-GlcNAc and F16BP in *ppg1Δ* cells after 18 hours of growth in SD medium:

**Given that all the experiments conducted in this manuscript were performed using YPD medium, and YPD is better reflective of a complex nutrient medium that natural yeasts would be exposed to (and more relevant to adaptation in changing nutrient sources), we feel that the manuscript remains more readable and relevant in YPD (complex medium).   Incorporating these data from minimal medium in the manuscript would disrupt its coherence and the overall flow. In addition, the very elaborate estimates of carbon flux through gluconeogenesis, using ^13^C label tracking, have been done in this medium only. As noted earlier, this is the first study (to our knowledge) to do this in the context of any phosphatase regulating gluconeogenic flux. Repeating the entire study in minimal, defined medium is therefore impractical. However, we have included these data for the reference of the reviewer, and believe it addresses the primary concerns.**

3) While loss of PP1G does not affect growth on glucose, cells entered post-diauxic shift show some latency, suggesting that they would resume more slowly on gluconeogenic substrates, which is mainly ethanol. Thus, it might be relevant to check whether this Ppase is not important growth on gluconeogenic substrate, such as ethanol and acetate (not glycerol at least if using mineral synthetic medium such as YNB as this is not a good substrate), and clearly do this minimal medium (YNB) to get rid of other carbon substrates.

Our results (included in the manuscript) indicate that the *ppg1Δ* cells show reduced growth in the post-diauxic phase. We carried out a shift experiment to investigate if these cells resume growth slowly when shifted to gluconeogenic substrates. We cultured *ppg1Δ* cells in glucose-replete conditions and shifted them to a medium with ethanol as sole carbon source. As anticipated, we observed that the *ppg1Δ* cells resumed growth at a slower rate in ethanol containing medium.

Data showing the growth dynamics of *ppg1Δ* cells after shift to ethanol containing medium:

**Given that all the experiments conducted in this manuscript were performed using YPD medium, and no shift experiments were included (as explained earlier), we believe that incorporating this data in the manuscript could disrupt its coherence, cause confusion to readers, and disrupt the overall flow. However, we include this for the reviewer, to address this point, but would prefer to not include it in the manuscript.**

4) Technical methods for quantifying intracellular metabolites are missing! There is a link to a paper from the same authors that is even not accessible! Measuring intracellular metabolites is very tricky as how quenching, sampling and extraction have been made are critical to get reliable data.

We apologize for this. Having studied trehalose and flux towards this for so long (e.g. see PMID: 32876564, PMID: 31758251, PMID: 27090086, and PMID: 31241462), we inadvertently took for granted some of the details of these methodologies. We have extensively described many ways to quantitatively estimate trehalose production and *flux* in this methods paper PMID: 32181267 (also see some other references particularly PMID: 32876564, PMID: 31758251, PMID: 27090086, and PMID: 31241462. **We have now modified the methods section and mentioned the detailed extraction protocols and methods to measure trehalose (Line 668).**

*“The metabolite extraction and analysis were carried out following protocols described in (Walvekar et al, 2019). For each experiment, 10 OD_600_ cells were used for metabolite extraction. First, the cells were quenched for 5 minutes in 60% methanol (maintained at -45^o^C). After centrifugation, the cell pellet was resuspended in the extraction buffer (75% ethanol) and kept at 80^o^C followed by incubation on ice and centrifugation. The supernatant was collected, dried, and then stored at -80^o^C till further use.”*

In addition, we have now included all the mass spectrometry parameters, as well as all the mass spectrometry raw data values as supplementary tables, so that any reader can analyse and quantify these metabolites.

5) Taking into account metabolites levels reported, the 3 to 4 fold levels of G6P and UDPGlc can account for higher capacity of trehalose accumulation because the trehalose synthase (TPS) displays Km that are in mM range for these metabolites and thus any increase of these metabolites will increase rate of TPS (old publication by {Vandercammen, 1989 #3278;Londesborough, 1993 #3899}

Thanks for this note. Indeed, a major idea that is nucleated by our study is the possible roles of mass action based control of gluconeogenic flux. This is also related to some of our responses included earlier. **We have now contextually discussed the importance of mass action-based regulation in the discussion section, and included key references. Included in the discussion (Line 466).**

*“This function of Ppg1, as uncovered in this study, differs from regulation mediated by related phosphatases. How might this occur? An underappreciated but important mediator of metabolic adaptation is the direct modulation of metabolic outputs or flux, through a combination of mass action and allosteric regulation (and without invoking transcriptional changes). Even in unicellular organisms like S. cerevisiae, over 50% of metabolic regulation occurs through such mechanisms (Hackett et al, 2016). In this study, the loss of Ppg1 increases the levels of gluconeogenic intermediates, precursors of cell wall and storage carbohydrates (Fig. 2A). Increasing flux towards G6P and UDP-glucose would be one way of supporting the increased synthesis of storage carbohydrates without requiring alterations in enzyme levels, driven primarily by mass action. Classic studies of the trehalose synthesis enzymes in yeast (Vandercammen et al, 1989; Londesborough & Vuorio, 1993) indicate this possibility.”*

6) 13C-labelling indicates a higher GNG flux in a pp1gD strain. Thus, one might expect faster growth resumption, which is the opposite that what is observed in a pp1g deletion strain? How to reconcile these data?

In the post-diauxic phase, the *ppg1Δ* cells exhibit increased gluconeogenic flux, suggesting an imbalanced carbon allocation. However, this increased gluconeogenic flux need not necessarily support better adaptation on gluconeogenic substrates. What is really important to a cell is the balance of allocation of carbon resources (discussed more extensively in a recent study from our lab: (Rashida *et al,* 2021 PMID: 33853774), which we now contextually cite here). In *ppg1Δ* cells, this imbalance in carbon allocation results in increased consumption of amino acids towards gluconeogenic outputs and might limit their availability for other cellular processes resulting in reduced growth and biomass production. Hence, even though the gluconeogenic flux is higher in *ppg1Δ* cells, these cells have reduced growth in post-diauxic phase. Achieving a precise equilibrium of flux towards different outputs is crucial for optimum growth or appropriate adaptation. This is an interesting and non-intuitive point, hence we now more extensively discuss this in the manuscript. **Included in the discussion (Line 477).**

***“****This increase in gluconeogenic flux in ppg1Δ cells indicates an imbalance in carbon allocations, resulting in increased consumption of amino acids towards gluconeogenic outputs, and therefore might limit their availability for other cellular processes. Hence, even though the gluconeogenic flux is higher in ppg1Δ cells, these cells have reduced growth in the post-diauxic phase. This plausible mode of regulation via Ppg1 could be systematically investigated in future studies, as an example of regulation mediated via some combination of mass action, concentration, allostery and enzyme regulation. These additional mechanisms (through scaffolding systems working together with signaling systems) to mediate overall metabolic outputs might be more prevalent than currently appreciated. In this context, we recently identified a signaling axis with Snf1 (AMPK) and TORC1 (via Kog1) in enabling precise carbon allocations, ensuring optimum growth and adaptation during nutrient limitation (Rashida et al, 2021).****”***

7) Sensibility of mutant cells to CR is borderline. Could you confirm with Calcofluor white which usually is more sensitive to minor cell wall modification, and notably when chitin is increased

This is a good suggestion. We studied the growth of *ppg1Δ* cells in the presence of Calcofluor white and observed a similar growth defect as seen with CR, but the images are very clear. Some of this is a reflection of the nature of our light-box black-and white camera (and visibly and in color, the plates look much better!). **We have now added this data in Fig S2D and mentioned this in the text (Line 206).**

*“Increased chitin accumulation is known to sensitize cells to cell wall stress (Ram & Klis, 2006; Vannini et al, 1983); hence, we studied the growth of ppg1Δ cells in the presence of two cell wall stress agents, Congo red and Calcofluor white. Expectedly, (and as observed earlier (Hirasaki et al, 2010)) the growth of ppg1Δ cells was reduced in the presence of either Congo red or Calcofluor white (Figure S2C, D).”*

Data showing the growth of *ppg1D* cells in the presence of Calcofluor white (now new Fig. S2D):

8) Is there any idea about the phosphorylation site on far11. I did not check on the phosphoproteomes data, but this might be worth to do and in that case, the loss of this phosphorylation shall be similar to loss of pp1G (no necessary to do that in this report)

Addressed extensively in the section 2 of this document.
