# Supplementary material for: The PP2A-like phosphatase Ppg1 mediates assembly of the Far complex to balance gluconeogenic outputs and enables adaptation to glucose depletion
Source: PLoS Genet. 2024 Mar 7;20(3):e1011202. doi: 10.1371/journal.pgen.1011202 (PMC10950219; doi:10.1371/journal.pgen.1011202)
Supplement: Attachment — Submitted filename: Response letter.docx [file pgen.1011202.s014.docx]

We thank the editor and the reviewers for the detailed feedback on our manuscript after our previous revision, where the reviewers appreciated the substantial changes that had been made. We now include a further revised manuscript, where we fully address the last standing query from one reviewer (regarding the identification of specific phosphorylation sites on Far11), through an additional series of direct experiments. The revised manuscript now address this remaining query, and incorporates these changes. The revised manuscript includes both new experimental data, as well as text reflecting the same.

Summarizing the changes in this revision we have included a new results section, which presents new data on the identification of specific ser/thr residues on the Far11 protein that are phosphorylated in a Ppg1 dependent manner, and the extent of regulation of Far11 phosphorylation by Ppg1. Additionally, we have included an extra figure, with new Figure 3, mainly illustrating the Ppg1-mediated regulation of Far complex assembly.

A detailed response to the reviewer’s comments is included below.

In the response letter below as well as the revised manuscript, we have addressed the concerns raised by reviewer 2. Our responses to queries are provided in blue text. Additionally, a fully marked up version of the manuscript (with the incorporated changes) has also been uploaded.

**Point-by-point response to reviewer comments:**

Reviewer #2:

Specific comments

1.Figure 3E: Why does the molecular weight of Far11-FLAG after CIP treatment differ between the Ppg1-H11N and Ppg1 strains? Both should be fully dephosphorylated; it is possible that modifications other than phosphorylation may have occurred by Ppg1.

We add some additional clarifications to this point. The difference in mobility of Far11 in wild-type and Ppg1^H111N^ cells can certainly be because of post-translational modifications other than phosphorylation. However, these modifications are also regulated in a Ppg1-dependent manner. In response to the next point (Comment 3), we have now completed a comprehensive proteomics analysis of Far11. Through this analysis, we identify multiple phosphorylation sites on Far11 (Ppg1 dependent) and also do not identify any post-translational modifications other than phosphorylation. These are extensively clarified in detail in comment 3. **We now include this in the text (Line no. 294-297):**

*“At this stage, while these experiments are consistent with a role of dephosphorylation in Far11 function and the assembly of the Far complex, these do not preclude other post-translational modifications in addition to phosphorylation.”*

2. Figure 4A-C: In the presence of Far10, some Far complexes can localize to both mitochondria and ER. Even if the amount is undetectable by fluorescence microscopy, the possibility that it affects the phenotype cannot be ruled out.

That is a fair point. In Mito-Far and ER-Far cells, the Far10 protein, with its intact TA domain, may indeed localize to the surface of both organelles. Therefore, in order to determine if the Far complex in Far9-Tom5 and Far9-Cyb5 strains is specifically confined to the mitochondria and endoplasmic reticulum, we deleted the tail anchor domain of Far10 in cells with these respective genetic backgrounds. Using these strains, we now confirm using fluorescence microscopy that the localization of Far complex is restricted to the specific compartments. Further, we also assessed Far complex function in this Far10 TA domain deleted strain using a growth assay in the presence of Congo red. We find that the deletion of the tail anchor domain of Far10 in both genetic backgrounds *does not* impact the subcellular localization and function of the Far complex. In summary, these findings reiterate that the specific subcellular localization of the Far complex is not crucial for its overall function, as long as it is tethered to either the ER or mitochondrial surface. **We now include this new result in the text (Line no. 384-394):**

*“*Additionally, in the Mito-Far and ER-Far cells, the Far10 protein (which has an intact TA domain) can localize to the surface of both organelles. To ascertain whether the Far complex in Far9-Tom5 and Far9-Cyb5 strains is exclusively localized to the mitochondria or endoplasmic reticulum (respectively), we deleted the tail anchor domain of Far10 in these genetic backgrounds. We generated Far9-Cyb5/Far10ΔTA and Far9-Tom5/Far10ΔTA strains, and assessed their subcellular localization. Far9-Cyb5/Far10ΔTA and Far9-Tom5/Far10ΔTA cells showed exclusive ER or mitochondrial localization, respectively (Figure S5B). To study the Far complex function, we assessed growth of these cells in presence of Congo red. Both these strains phenocopied wild-type cells and were not sensitive towards Congo red (Figure S5C)*.”*

The data confirming the localization of Far complex in Far9-Cyb5/Far10ΔTA and Far9-Tom5/Far10ΔTA cells is now new Figure S5B, and is shown below:

The data showing the growth of WT, *ppg1Δ*, Far9-Cyb5 Far10ΔTA and Far9-Tom5 Far10ΔTA cells in the presence of Congo red (now new Figure S5C):

3. it is necessary to prove the model that Ppg1 dephosphorylates Far11 and the dephosphorylated Far11 forms Far complex. As shown in comment 4 of first round, identification of the phosphorylation site of Far11 would be the easiest way, but other methods are also acceptable.

We accepted this challenging request by the reviewer, and performed a series of direct experiments. We first purified Far11 from WT and Ppg1-catalytically dead cells, with multiple biological replicates, and used proteomic analysis (LC-MS/MS) to pinpoint specific phosphorylation sites in Far11 that are regulated by Ppg1. The detailed methodology for this experiment is now mentioned in the updated methods section (Line no: 707-720). In our earlier response, we had indicated the possibility that, as is typically the case for PP2A-family phosphatase targets, Far11 might have multiple phosphorylation sites. This has indeed proven to be the case. Through our new studies, we now unambiguously identify *multiple* ser/thr residues on Far11 that are Ppg1 dependent. Several phosphorylation sites on Far11 show increased abundance in the Ppg1 catalytically inactive (Ppg1^H111N^) cells, suggesting dephosphorylation in a Ppg1 dependent manner. In our analysis, we found ~8 phospho-serine residues increase in Ppg1^H111N^ cells, while three ser/thr sites are not altered in these cells. Additionally, there remain several ser/thr sites on Far11 that we could not specifically identify. Thus, substantially adding to the extensive biochemical data provided earlier, we now provide direct evidence of Ppg1 dependent Far11 dephosphorylation, on multiple ser/thr residues on Far11. We now propose that the Ppg1 dependent dephosphorylation of these multiple phosphorylated residues on Far11 facilitates Far11 interacting with Far8 to complete the Far complex assembly.

Given the extensive nature of these experiments, an entire new section has now been added to the manuscript.

**The relevant sections of the text now read (lines 299-334):**

***“Ppg1 regulates the phosphorylation status of multiple ser/thr residues in Far11***

*A closer inspection of the Far11 sequence identifies over 19 ser/thr residues in Far11 that may be putatively phosphorylated (Fig 3F), based on prediction algorithms [56]. We therefore directly addressed the identification of Far11 ser/thr residues that are regulated by Ppg1. For this, we first immunopurified sufficient amounts of Far11 from post-diauxic WT and Ppg1^H111N^ cells (Fig 3G). Subsequently, immunopurified fractions were resolved on SDS-PAGE gels, the bands corresponding to Far11 were excised, and in-gel digestion was carried out. The phosphorylated residues of Far11 were identified using LC-MS/MS. This LC-MS/MS based analysis was able to identify peptides that collectively covered ~60% of Far11 protein sequence. Notably, this analysis unambiguously identified ~8-12 phosphorylated ser/thr sites on Far11, across four independent replicates (respectively of WT and Ppg1^H111N^ samples) (Fig 3H). From this analysis, we observed changes in the relative phosphorylation status of multiple serine residues in Ppg1^H111N^ cells, while some residues did not show any changes in phosphorylation (Fig 3I).*

*We classified these phosphosites based on their regulation by Ppg1 into three groups. The first group includes high-confidence sites, which show a consistent increase in phosphopeptide abundance across all four replicates of Far11 isolated from Ppg1^H111N^ cells (Fig 3J). This included residues pS81, pS109, pS110, and pS528, which all showed a significant increase in Ppg1^H111N^ cells, suggesting increased phosphorylation of these serine residues (Fig 3I, 3J, S3B). This would be consistent with reduced dephosphorylation of these residues in the catalytically inactive Ppg1^H111N^ cells. The second group consisted of lower-confidence hits of phosphopeptides found in some biological replicates of Ppg1^H111N^ cells. Notably, residues pS457, pS489, pS503, and pS690 showed an increase in some replicates of Ppg1^H111N^, or were absent in WT samples (Fig S3B). This group of ser/thr residues are putatively regulated by Ppg1, but this identification is not unambiguous, due to technical limitations in mass spectrometry based detection. The third group included residues pT520, pS527, and pS939 which did not show any change in relative phosphorylation status in Ppg1^H111N^ vs WT cells, indicating they are not under the regulation of Ppg1 (Fig 3I, 3J, S3B).*

*In summary, our analysis identified several, specific ser/thr residues in Far11 that undergo dephosphorylation in a Ppg1 dependent manner (Fig 3K). Collectively, our data now find that Ppg1 regulates the phosphorylation of multiple Far11 ser/thr residues, and this dephosphorylation of Far11 (in a Ppg1 dependent manner) is necessary for the assembly of the Far complex.”*

This data showing the regulation of Far11 phosphorylation by Ppg1 is now new Figures 3F-3K, as well as a new Figure S3B, and Supplementary Table 3 with complete phosphoproteomics data:

(also see the new Figure S3B):
